# Comparison of Functional Vision and Eye-Related Quality of Life between Myopic Children Treated with Orthokeratology and Single-Vision Spectacles in Southern China

**DOI:** 10.1155/2023/7437935

**Published:** 2023-04-08

**Authors:** Tingting Yang, Rongsheng Hu, Wen Tian, Ying Lin, Yamei Lu, Xiaolin Liang, Danying Zheng, Xinyu Zhang

**Affiliations:** ^1^Department of Ophthalmology, First Affiliated Hospital of Jinan University, Jinan University, Guangzhou, China; ^2^Department of Ophthalmology, The Sixth Affiliated Hospital of Guangzhou Medical University, Qingyuan People's Hospital, Qingyuan, China; ^3^Department of Urology, The Sixth Affiliated Hospital of Guangzhou Medical University, Qingyuan People's Hospital, Qingyuan, China; ^4^Qingyuan Aier Eye Hospital, Qingyuan, China; ^5^State Key Laboratory of Ophthalmology, Zhongshan Ophthalmic Center, Sun Yat-Sen University, Guangdong Provincial Key Laboratory of Ophthalmology and Visual Science, Guangdong Provincial Clinical Research Center for Ocular Diseases, Guangzhou, China

## Abstract

**Objective:**

To compare eye-related quality of life between Chinese children wearing orthokeratology (OK) contact lenses and single-vision spectacles (SVS) using the Pediatric Eye Questionnaire (PedEyeQ) and to evaluate the impact of different myopia correction methods on children and their parents.

**Methods:**

Children aged 12–17 years and their parents/legal guardians were recruited. The children's myopia ranged from −0.50 to −5.00 diopters (D), and their astigmatism was <1.50 D. They had all been wearing OK contact lenses or SVS for at least 12 months. The children completed the Child PedEyeQ. One of their parents (or a legal guardian) completed the Proxy PedEyeQ and the Parent PedEyeQ. Rasch-calibrated PedEyeQ scores were calculated for each domain and were converted to a scale from 0 to 100 for statistical analysis.

**Results:**

A total of 50 children wearing OK contact lenses, 43 children wearing SVS, and their parents/legal guardians completed the questionnaires. The scores of all Child, Proxy, and Parent PedEyeQ domains in the OK contact lens group were higher than those in the SVS group (all *P* < 0.05). In the mild and moderate myopia subgroups, the Child, Proxy, and Parent PedEyeQ scores in the mild myopia OK contact lens subgroup were higher than those in the mild myopia SVS group (all *P* < 0.05) except functional vision and bothered by eyes/vision domains for the proxy PedEyeQ. Similarly, the Child, Proxy, and Parent PedEyeQ scores in the moderate myopia OK contact lens subgroup were higher than those in the moderate myopia SVS subgroup (all *P* < 0.05) except impact on parent and family domain for the parent PedEyeQ. In the subgroup analysis of glasses type, no significant score difference of any Child, Proxy, and Parent PedEyeQ domain was detected between mild and moderate myopia in either the OK contact lens group or the SVS group (all *P* > 0.05).

**Conclusion:**

Compared with children wearing SVS, children wearing OK contact lenses have better functional vision and eye-related quality of life. Moreover, OK contact lens has a better correction effect, higher acceptance rates, and less impact on parents and families than SVS.

## 1. Introduction

Myopia is one of the most common eye diseases in the world [1–3], with a prevalence of about two billion people, accounting for 28.3% of the global population [4]. In China, the prevalence of myopia is more than 60% among 12-year-old children, nearly 80% among 16-year-old individuals, and more than 90% among college students [5–7]. Myopia and correction methods have a significant impact on patients' visual quality and related quality of life [8–10]. Children's visual quality of life is also affected by family factors. [11]. Therefore, investigating the children's eye-related quality of life and evaluating the effect of different myopia correction methods on proxy or parents can greatly help the doctors' myopia correction decisions.

At present, the nonsurgical correction of myopia in children is mainly achieved by wearing lenses, single-vision spectacles (SVS), and orthokeratology (OK) contact lenses [12]. Santodomingo-Rubido et al. used the Pediatric Refractive Error Profile (PREP) questionnaire to compare visual quality of life between children wearing OK contact lenses and SVS and found that in most items, the scores of children wearing OK contact lenses were significantly higher than those of children wearing SVS, except for near vision and handling of optical corrections [13]. In a study of Chinese children, Yang et al. used the same questionnaire to compare the two correction methods and reached similar conclusions [14]. PREP is a tool especially used to measure the quality of life of children receiving refractive correction [15], and the questionnaire related to vision-related quality of life includes National Eye Institute-Refractive Quality of Life (NEI-RQL), Self-Perception Profile for Children Global Self-Worth scale (S-PPCGS-W), Study-specific Questionnaire, Quality of Life Impact of Refractive Correction (QIRC), Orthokeratology and Contact Lens Quality of Life Questionnaire (OCL-QoL), and National Eye Institute-Visual Function Questionnaire (NEI-VFQ) [16]. These tools are generally used to evaluate functional vision or quality of life under specific eye conditions and only allow children or parent's reporting but not allow the reporting from both of them.

The Pediatric Eye Questionnaire (PedEyeQ), developed by Hatt et al. in 2019, can be used to evaluate the functional vision and eye-related quality of life of children of any age and with any eye condition [17]. Moreover, it can be used to evaluate children and parents/legal guardians at the same time. The PedEyeQ has been applied to and verified in a wide range of pediatric eye diseases, such as strabismus, amblyopia, ametropia, cataract, and retinal diseases [17–25], but has not been used in a comparative study of OK contact lenses and SVS worn by myopic children. Thus, this study used the PedEyeQ to compare functional visual acuity and eye-related quality of life between Chinese myopic children aged 12–17 years wearing OK contact lenses and SVS and the impact of different myopia correction methods on parents and families to serve as a guide for doctors' choices of correction methods.

## 2. Materials and Methods

### 2.1. Subjects

Subjects were randomly recruited from the Qingyuan People's Hospital of Guangdong Province and Aier Eye Hospital in Qingyuan, China. Following previous studies, patients aged 12–17 years with myopia of −0.50 (D) to −5.00 diopters (D) and refractive errors with astigmatism (rule) of <1.50 D [26] who had been wearing OK contact lenses (for at least 8 h per night) or SVS for at least 12 months and had no systemic or ocular diseases were included. The children and their parents/legal guardians filled out the PedEyeQ on-site after routine eye examinations. Myopia was defined as spherical equivalent (SE) refraction worse than −0.5 D in at least one eye. Mild myopia was defined as −3.00 D ≤ SE ≤ −0.5. Moderate myopia was defined as −6.00 D < SE ≤ −3.25 D [27, 28].

This study followed the principles of the Declaration of Helsinki. Informed consent was obtained from the children and their parents/legal guardians before data collection.

### 2.2. PedEyeQ

The PedEyeQ [17] includes the Child, Proxy, and Parent questionnaires and includes three versions: for ages 0–4, 5–11, and 12–17. The version used in this study was for ages 12–17. The Child PedEyeQ consists of four individually scored domains: functional vision, bothered by eyes/vision, social, and frustration/worry. The Proxy PedEyeQ consists of five domains: the four Child PedEyeQ domains and the eye care domain. The Parent PedEyeQ consists of four domains: impact on parent and family, worry about the child's eye condition, worry about the child's self-perception and interactions, and worry about the child's functional vision. The children filled out the Child PedEyeQ themselves, and one of their parents (or their legal guardians) filled out the Proxy PedEyeQ and Parent PedEyeQ. The responses to all items were “never,” “sometimes,” or “always.” Questionnaires, scoring algorithms, and lookup tables are freely available at https://public.jaeb.org/pedig/view/Other_Forms.

### 2.3. Statistical Analysis

Statistical analysis was performed using Stata 15.0 software (StataCorp, College Station, TX, USA). For each subject, Rasch scores were calculated for each PedEyeQ domain using a previously published Rasch lookup table (https://public.jaeb.org/pedig.) and then converted to a scale from 0 (worst) to 100 (best) for interpretation. The main analysis compared the Child, Proxy, and Parent PedEyeQ scores of children wearing OK contact lenses to those of children wearing SVS. The patients were further divided into mild and moderate myopia subgroups according to the degree of myopia and type of glasses worn, and comparisons of the Child, Proxy, and Parent PedEyeQ scores were performed between the subgroups within and between the OK contact lens and SVS groups. Kruskal–Wallis test was used to compare the difference of scores between groups and subgroups, and *P* < 0.05 was considered statistically significant.

## 3. Results

A total of 93 children—50 in the OK contact lens group and 43 in the SVS group—and their parents/legal guardians were included in this study.  Table 1 summarizes the demographic characteristics and ocular baseline data of the children in both groups. There were no statistically significant differences in demographics between the two groups (all *P* > 0.05). Likewise, the two groups did not differ significantly in terms of ocular baseline data (all *P* > 0.05), except for the left cylinder, which was significantly higher in the OK contact lens group than in the SVS group (*P* < 0.05).

Table 2 shows the Child, Proxy, and Parent PedEyeQ domain scores of the OK contact lens and SVS groups. The scores of the OK contact lens group were significantly higher than those of the SVS group in all domains of the three PedEyeQ versions (all *P* < 0.05).

**Figure 1:**
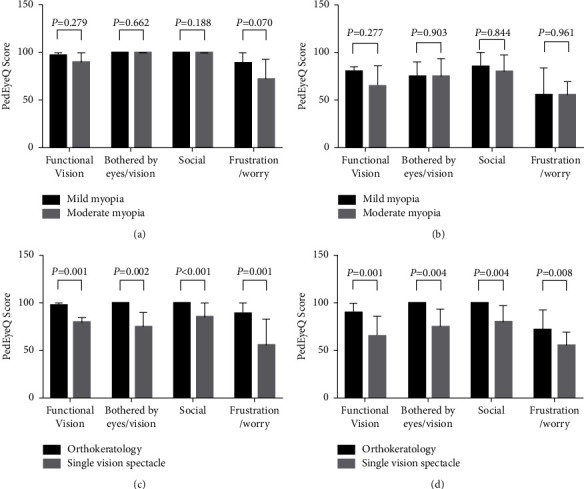
Child PedEyeQ domain scores of the mild and moderate myopia subgroups in the OK contact lens and SVS groups.

Figure 1 shows the Child PedEyeQ domain scores of the mild and moderate myopia subgroups in the OK contact lens and SVS groups. As shown in Figure 1(a), there were no statistically significant differences in any domain scores between the mild and moderate myopia subgroups of the OK contact lens group (all *P* > 0.05). Likewise, no statistically significant differences were observed in any domain scores between the mild and moderate myopia subgroups of the SVS group (all *P* > 0.05) (Figure 1(b)). However, as shown in Figure 1(c), the mild myopia OK contact lens subgroup had significantly higher scores in all domains than the mild myopia SVS subgroup (all *P* < 0.05). Likewise, the scores of the moderate myopia OK contact lens subgroup were significantly higher than those of the moderate myopia SVS subgroup in all domains (all *P* < 0.05) (Figure 1(d)).

**Figure 2:**
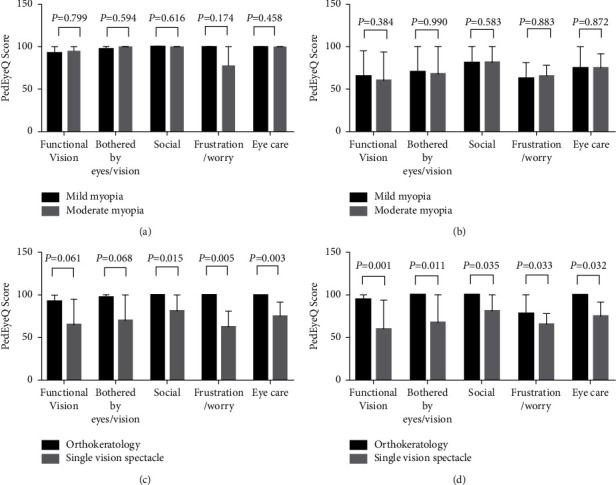
Proxy PedEyeQ domain scores of the mild and moderate myopia subgroups in the OK contact lens and SVS groups.

Figure 2 shows the Proxy PedEyeQ domain scores of the mild and moderate myopia subgroups in the OK contact lens and SVS groups. As shown in Figure 2(a), there were no statistically significant differences in any domain scores between the mild and moderate myopia subgroups of the OK contact lens group (all *P* > 0.05). Likewise, there were no statistically significant differences in any domain scores between the two subgroups in the SVS group (all *P* > 0.05) (Figure 2(b)). However, as shown in Figure 2(c), the mild myopia OK contact lens subgroup had significantly higher scores than the mild myopia SVS subgroup in all domains except for the functional vision and bothered by eyes/vision domains. Similarly, the scores of the moderate myopia OK contact lens subgroup were significantly higher than those of the moderate myopia SVS subgroup in all domains (all *P* < 0.05) (Figure 2(d)).

**Figure 3:**
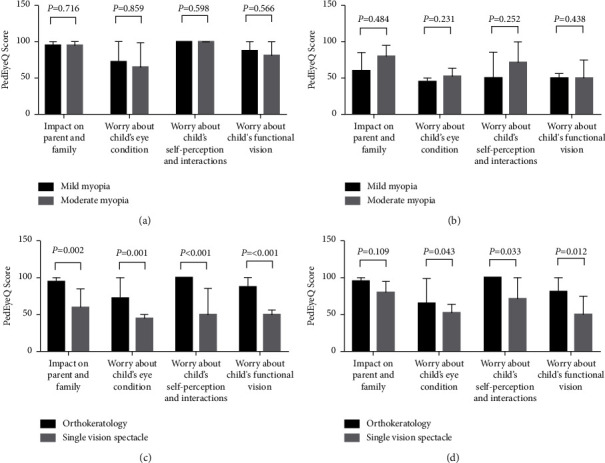
Parent PedEyeQ domain scores of the mild and moderate myopia subgroups in the OK contact lens and SVS groups.

Figure 3 shows the Parent PedEyeQ domain scores of the mild and moderate myopia subgroups in the OK contact lens and SVS groups. There were no differences in any domain scores between the mild and moderate myopia subgroups of the OK contact lens group (all *P* > 0.05) (Figure 3(a)). Likewise, as shown in Figure 3(b), no differences were observed in any domain scores between the mild and moderate myopia subgroups of the SVS group (all *P* > 0.05). However, the mild myopia OK contact lens subgroup had significantly higher scores than the mild myopia SVS subgroup in all domains (all *P* < 0.05) (Figure 3(c)). Similarly, in the moderate myopia group, OK contact lens subgroup had significantly higher scores than the SVS subgroup in all domains except the impact on parent and family domain.

## 4. Discussion

In this study, the OK contact lens group had significantly higher scores than the SVS group in all domains of the Child, Proxy, and Parent PedEyeQ. Moreover, the mild myopia OK contact lens subgroup had significantly higher scores than the mild myopia SVS subgroup in all domains of the three PedEyeQ versions, except for the functional vision, and bothered by eyes/vision domains of the Proxy PedEyeQ. Similarly, the moderate myopia OK contact lens subgroup had significantly higher scores than the moderate myopia SVS subgroup in all domains of the three PedEyeQ versions, except for the impact on parent and family domain of the Parent PedEyeQ. Conversely, the glasses type subgroup analysis showed no statistically significant score differences in any domains of the three PedEyeQ versions between the mild and moderate myopia subgroups in either the OK contact lens group or the SVS group.

The OK contact lens group in this study had significantly higher scores than the SVS group in the Child and Proxy PedEyeQ functional vision domain, which mainly assesses children's vision, learning, concentration, schoolwork, sports, and difficulties encountered. This result is in line with previous studies reporting that children wearing contact or OK contact lenses have better far and overall vision than children wearing SVS [13–15, 29]. Best corrected visual acuity (BCVA) did not differ significantly between the two groups in this study. However, BCVA can reflect only central vision. Studies have shown that OK contact lenses can provide not only better central vision but also better peripheral vision than SVS and can improve adjustment accuracy [30–33]. This may explain the higher functional vision scores in the OK contact lens group. The scores of the Proxy PedEyeQ functional vision domain, which focuses on the child's daily activities and school-related tasks, did not differ significantly between the mild myopia OK contact lens subgroup and the mild myopia SVS subgroup. This may be because of some parents' belief that mild myopia has a lesser impact on children's daily lives and learning, even without refractive correction.

The OK contact lens group in this study also had significantly higher scores than the SVS group in the Child and Proxy PedEyeQ bothered by eyes/vision, social, frustration/worry, and eye care domains, which primarily assess children's vision-related quality of life. This is consistent with previous research showing that children wearing OK contact lenses score higher on appearance, satisfaction, activity, and peer perception than children wearing SVS [13, 14, 34, 35]. OK contact lenses are worn only at night, allowing children to enjoy clear vision during the day, making it more convenient for them to participate in activities with friends and family, and increasing their satisfaction with their appearance. Thus, children avoid the distress associated with poor daytime vision requiring the use of glasses, thereby enhancing socialization. Research also suggests that OK contact lens has favorable effects on children's quality of life, behavior, and psychology [36]. Furthermore, OK contact lens not only corrects myopia but also safely and effectively controls myopia progression [26, 37–39], which may make children and parents less concerned about worsening eye condition. The higher scores of the OK contact lens group in the Proxy PedEyeQ eye care domain are in line with the findings of Pomeda et al., who found that MiSight contact lenses were associated with higher handling scores than SVS [29]. Conversely, Yang et al. reported that children wearing OK contact lenses had significantly lower scores related to optical corrective handling aspects [14], while Santodomingo-Rubido et al. found that children wearing OK contact lenses and children wearing SVS had similar handling scores [13]. Although handling OK contact lenses undeniably requires more time and effort than handling spectacles, OK contact lenses are used and removed at home, and all lens handling can be performed under parents' supervision. Moreover, research suggests that children are able to handle contact lenses successfully [40]. It should be noted, however, that all children involved in this study were aged 12–17 years and had the ability to handle lenses without their parents' assistance. The subgroup analysis showed no significant differences in Proxy PedEyeQ bothered by eyes/vision domain scores between the mild myopia OK contact lens subgroup and the mild myopia SVS subgroup. Again, this may be related to some parents' belief that mild myopia has a minimal impact on children's daily lives and learning.

The OK contact lens group in this study scored significantly higher than the SVS group in all Parent PedEyeQ domains, which primarily assess parents' concerns about children's functional vision, self-perception and interactions, and eye condition and its effects on family-specific aspects. Research suggests that parents' own quality of life can be affected by their children's vision problems [23]. Chang et al. reported that myopia control and convenience of outdoor activities were important reasons parents opted for OK contact lenses for their children [41]. The reason that the parents in the OK contact lens group reported less concern about their children's eyes than parents in the SVS group may be related to their understanding that OK contact lenses are currently the most effective means of controlling myopia progression [37] and to the fact that there is no need to wear glasses during the day, which facilitates children's learning, living, and exercise. In contrast, SVS only correct myopia and do not address the underlying problem of axial growth and myopic progression, thus often requiring periodic (6–12-month) reassessment of refraction and lens replacement to restore normal vision. Failure to correct myopia promptly can worsen myopic progression by up to 30% [42–44], which may be a major contributor to concerns about children's eye condition among parents of children wearing SVS. The Parent PedEyeQ impact on parent and family domain scores in this study did not differ significantly between the moderate myopia OK contact lens group and the moderate myopia SVS group, perhaps because most children with moderate myopia wore SVS for a long time, and their parents were accustomed to it.

A main limitation of this study was that all subjects were from the same city. Thus, the generalizability of the results may be limited, as findings may vary by race/ethnicity and other demographic factors. Multicenter studies with larger samples are needed to validate our results. The strength of this study was that we applied the PedEyeQ to compare functional vision and eye-related quality of life between myopic children wearing OK contact lenses and those wearing SVS and assessed the impact of different myopia correction methods on parents and families. As parents play an active role in deciding what myopia correction modality their children choose, understanding their thoughts can help eye care professionals provide children with the appropriate modality.

## 5. Conclusion

This study suggests that Chinese children wearing OK contact lenses have better overall functional vision and eye-related quality of life than those wearing SVS and that OK contact has a lesser impact on their families. Furthermore, the proven safety [45] and effectiveness of OK contact lens in controlling myopia progression [46] suggest that practitioners should preferentially consider it for the clinical management of visual acuity correction in children.

## Figures and Tables

**Table 1:** Demographic characteristics and ocular baseline data of the participants.

	OK	SVS	*P* value
Age (year)	14 (13, 16)	14 (13, 15)	0.919
Male (%)	26 (52.0%)	23 (53.5)	0.886
Spherical power of right eye (D)	−3.0 (−4.25, −2.0)	−2.5 (−4.0, −2.0)	0.338
Spherical power of left eye (D)	−2.88 (−3.75, −1.94)	−2.75 (−3.75, −1.75)	0.717
Cylinder power of right eye (D)	0 (−0.5, 0)	0 (−0.5, 0)	0.346
Cylinder power of left eye (D)	−0.5 (−0.81, 0)	0 (−0.75, 0)	0.026
BCVA of right eye, logMAR	0 (0, 0)	0 (0, 0)	0.302
BCVA of left eye, logMAR	0 (0, 0)	0 (0, 0)	0.241
Duration of lens wear (months)	24 (18, 34.3)	30 (20, 38)	0.057

OK = orthokeratology; SVS = single-vision spectacles.

**Table 2:** PedEyeQ domain scores in orthokeratology and single-vision spectacles groups.

PedEyeQ domains	Median (range) PedEyeQ scores		*P* value	Mean difference (95% CI)
	OK (*N* = 50)	SVS (*N* = 43)		
Child				
Functional vision	92.5 (75.0, 100)	70.0 (50.0, 80.0)	<0.001	17.8 (10.8, 24.9)
Bothered by eyes/vision	100 (83.7, 100)	75.0 (50.0, 90.0)	<0.001	18.3 (10.4, 26.2)
Social	100 (90, 100)	85.0 (60, 100)	<0.001	17.1 (10.2, 24.1)
Frustration/worry	77.8 (61.1, 100)	55.5 (38.9, 72.2)	<0.001	19.9 (11.3, 28.5)
Proxy				
Functional vision	95.0 (80.0, 100)	65.0 (50.0, 95.0)	<0.001	16.9 (8.81, 24.9)
Bothered by eyes/vision	100 (82.5, 100)	70.0 (55.0, 100)	0.002	15.0 (5.96, 24.1)
Social	100 (87.5, 100)	81.3 (56.3, 100)	<0.001	13.7 (6.02, 21.4)
Frustration/worry	87.5 (67.2, 100)	62.5 (50.0, 81.3)	<0.001	16.6 (7.80, 25.3)
Eye care	100 (75.0, 100)	75.0 (50.0, 91.7)	<0.001	14.3 (6.76, 21.8)
Parent				
Impact on parent/family	95 (70.0, 100)	70.0 (50.0, 90.0)	<0.001	13.9 (4.99, 22.7)
Worry about child's condition	65.0 (50.0, 90.0)	45.0 (30.0, 60.0)	<0.001	22.4 (11.0, 33.9)
Worry about child's self-perception/interactions	100 (78.6, 100)	50.0 (50.0, 92.9)	<0.001	20.3 (10.7, 29.9)
Worry about child's visual function	87.5 (50.0, 100)	50.0 (37.5, 56.3)	<0.001	25.6 (14.1, 37.1)

OK = orthokeratology; SVS = single-vision spectacles; CI: confidence interval.

## Data Availability

The data used to support the findings of this study are available from the corresponding author upon request.
